# Distinguishing between those dying suddenly or not suddenly from coronary heart disease: long-term prospective results from the Northwick Park Heart Study

**DOI:** 10.1136/openhrt-2016-000440

**Published:** 2016-12-05

**Authors:** Tom Meade, Tim Clayton, Douglas Chamberlain

**Affiliations:** 1 Department of Non-Communicable Disease Epidemiology, London School of Hygiene and Tropical Medicine, London, UK; 2 Department of Medical Statistics, London School of Hygiene and Tropical Medicine, London, UK; 3 Brighton and Sussex Medical School, University of Sussex, East Sussex, Brighton, UK

## Abstract

**Aim:**

To establish whether ECG findings are associated with subsequent risk of sudden death from coronary heart disease (CHD).

**Methods and results:**

Potential risk factors for CHD were measured at entry to the first Northwick Park Heart Study of 2167 men. ECG findings were coded as high or low risk for CHD according to definitions in the Minnesota code. Sudden or non-sudden deaths were defined as occurring in less than or more than 24 hours, respectively. The only factor independently associated with sudden death among the 262 men dying of CHD was high-risk ECG. Of 184 sudden CHD deaths, 34 men (18.5%) had had high-risk ECGs at entry to the study compared with 5 (6.4%) of 78 men who experienced non-sudden deaths (adjusted OR 3.94 (95% CI 1.33 to 11.67)) (p=0.006). Findings were also compared among all 2167 men, where high-risk ECGs were again associated with sudden death. T-wave changes were the main abnormalities associated with a high risk of sudden death.

**Conclusions:**

In a group of men who had not previously experienced major episodes of CHD but who subsequently died from it, there was strong evidence that high-risk ECG changes, mainly T-wave abnormalities, differentiated between those who later died sudden deaths and those who survived for >24 hours.

**Key messages**

**What is already known about this subject?**

- T-wave abnormalities are associated with coronary heart disease (CHD) events and, if fatal, may be categorised as sudden (within 24 hours) or non-sudden. Some indirect comparisons for sudden deaths have been reported, identifying ventricular premature complexes, left ventricular hypertrophy and T wave changes as risk factors, for example. However there appear to have been few if any reports of direct comparisons based on data for risk factors and outcomes in the same analyses.

**What does this study add?**

- Results from direct comparisons are likely to be more accurate and therefore more useful than from indirect comparisons. This study provides strong evidence that among those initially free of major CHD, T-wave abnormalities are subsequently associated with sudden, but not non-sudden deaths due to CHD.

**How might this impact on clinical practice?**

- If confirmed in other studies, our results would provide good evidence for special surveillance of those with T-wave abnormalities. The possibility of identifying those likely to die suddenly if they show T-wave abnormalities may seem novel, even improbable. However, our results are strongly significant and consistent in different analyses and should therefore not be overlooked in encouraging further work to confirm or refute our findings on what is the most serious manifestation of CHD.

## Introduction

The 12-lead ECG is valuable not only in diagnosis but also as a predictor of future risk. There have been many studies of ECG risk factors for sudden death alone or for sudden and non-sudden events combined. One study of fatal and non-fatal coronary heart disease (CHD) found ventricular premature complexes predictive of fatal CHD, but no clear distinction between sudden and non-sudden CHD deaths.^1^ A further study found that left ventricular hypertrophy measured by 12-lead QRS voltage sum predicted individuals at an increased risk of sudden death^2^ but again there was no clear distinction between sudden and non-sudden deaths. However, findings in both studies were based on separate, indirect, comparisons of data for sudden and non-sudden deaths. Puddu *et al*^3^ identified ECG abnormalities as a risk factor for all-cause mortality over 40 years but this did not specifically assess sudden and non-sudden deaths. There appear to have been few if any studies making direct comparisons, that is, in the same analyses, of risk factors in those who die suddenly from CHD compared with those who survive acute episodes. This information might influence the planning of surveillance and investigation for those most likely to suffer sudden coronary death. This paper aims to determine whether details of the ECG can help identify those at an increased risk of sudden death.

## Methods

### Recruitment, investigations and follow-up

This report is based on data from the first Northwick Park Heart Study (NPHS-1),^4^ a conventionally designed prospective study carried out in three occupational groups in North-West London. From 1972 to 1978, NPHS-1 recruited 2167 men of all ages without previous episodes of myocardial infarction (MI).

Methods for clinical examination, laboratory measurements and assays have been described previously.^4^ Twelve-lead ECGs were carried out and classified according to the Minnesota code^5^ by an experienced coder. Each record was rated as low or high risk for a later major CHD event according to individual ECG items previously used to define risk in the British Medical Research Council's (MRC) trial of screening and treatment for mild hypertension.^6^ The MRC definitions include associations with CHD death (as well as non-fatal events) but do not specify whether fatal events were sudden or non-sudden. Table 1 presents the Minnesota code sections and the MRC high-risk components for fatal CHD within each Minnesota code section. For example, the abnormal codes 4_1-3_ and 5_1-3_ in section 4 of the Minnesota code (S–T junction depression) are those identified as high risk from the MRC classification. There were no abnormal MRC codes identified in Minnesota code sections 2 and 3. However, men on treatment for hypertension or with consistently raised blood pressure on remeasurements were not included in the MRC trial. This may largely explain why there were no high-risk MRC codes for left ventricular hypertrophy. Minnesota codes 6, 7 and 8 were not shown in the MRC publication, and high-risk ECG abnormalities were agreed by the authors. (In fact, these abnormalities were assigned to very few study participants.)

**Table 1:** High-risk MRC codes within specified Minnesota code sections

Minnesota code section	High-risk MRC code(s)
1. Q/QS pattern	1_1-2_
2. QRS duration	–
3. Left ventricular hypertrophy	–
4. ST junction and segment depression	4_1-3_, 5_1-3_
5. T-wave items, no ST depression	5_1-3_
6. AV conduction defect	6_1–5_
7. Ventricular condition defect	7_1_,7_4-6_
8. Arrhythmias	8_1–9_

Sections 2 and 3: no high-risk codes.

AV, atrioventricular.

Participants were followed up for up to 30 years. There were no losses to follow-up as the flagging of all participants in the National Health Service Central Register ensured that all deaths of study participants were automatically notified. On receipt of death notifications, further details were sought from general practitioners, hospitals and coroners. Information obtained in this way was then assessed according to WHO criteria^7^ by three doctors, independent of the study, who were blind to details such as smoking habit or laboratory results. These independent assessments were taken as the causes of death, as they occasionally differed from the certified causes. As many participants were elderly at the time of death, possible effects of multiple pathology need to be borne in mind in deciding about the causes of death. Information from the different sources was also used to determine the interval from onset of symptoms of a major CHD event to death.

Finally, one of the authors (DAC) assessed each ECG by current conventional diagnostic methods.

Three groups were established in classifying cases according to whether they were sudden or not.

Definite sudden coronary death was defined as death, often instantaneous, within 24 hours of onset of symptoms, when information from hospital records, coroners' reports or general practices enabled this definition to be made with certainty. Of 87 sudden deaths with known intervals, 71 (63%) occurred within 1 hour. (There were also 58 sudden deaths definitely occurring in <24 hours, but precise timings were not known.)

Non-sudden coronary death was recorded when there was clear evidence that coronary death had occurred >24 hours after the onset of symptoms.

Probable sudden coronary death was recorded when it was likely, though not certain, that death had occurred within 24 hours. An example would be a man seen alive one day but found dead in bed the next day, having died at an unknown time. From available but incomplete details, the large majority of deaths in this group were almost certainly sudden. Accordingly, we have, with one exception (see below), combined definite and probable sudden coronary deaths into a single group now referred to as sudden death. We may therefore have underestimated (but not exaggerated) any true differences between sudden and non-sudden deaths, to the extent that probable sudden deaths may include some that were non-sudden. One analysis has compared definite sudden deaths with definite non-sudden deaths (ie, omitting probable sudden deaths).

NPHS-I started in 1972, before the development of ethics committees in the UK. However, those approached about taking part in the study were given a full explanation of the reasons for it, its nature and what it would involve. The original study was funded by the British MRC (EPNCAC86).

### Statistical methods

Men were categorized into one of four groups according to cause of death (sudden CHD, non-sudden CHD, non-coronary death or alive at the end of follow-up.) A χ^2^ test was conducted to assess whether the percentage of men with a high-risk ECG differed across the four groups. Risk factors established as associated with death due to CHD as well as other potential confounding factors have been included in the analyses as previously described.^8^ They have been included in tables 3 and 4 irrespective of the strength of evidence for their association with sudden and non-sudden CHD mortality to more fully allow for any potential confounding. Categorisations of variables presented in the tables are consistent with those used previously as are the use of trend tests in the models where appropriate.^8^ All other variables detailed in the footnotes to the tables have been analysed as continuous variables except for alcohol use and social class, which have been treated as categorical variables.

Two approaches were used in order to assess whether there were any variables associated specifically with sudden when compared with non-sudden coronary deaths.

The first, a direct comparison between sudden and non-sudden death considered only the 262 men who died from CHD, to establish whether there were any differences in their risk profiles between men who experienced sudden or non-sudden deaths. Logistic regression was used with sudden death as the outcome. The main exposure of interest was the initial ECG finding for each participant. Univariable as well as age-adjusted analyses were also undertaken to compare results in the fully adjusted model. Since men were followed up for 30 years, a further analysis was undertaken, including age at death or end of follow-up in the multivariable model.

The second, an indirect comparison, included all 2167 men in the cohort prospectively followed up to establish whether associations of risk factors with CHD mortality differed between sudden and non-sudden coronary death. Two separate Cox regression models were fitted with (1) sudden coronary death as the outcome and (2) non-sudden coronary death as the outcome, again fitting all covariates of interest. These Cox models set the time scale to be current age while also including age at baseline as a covariate, to allow that baseline characteristics may have been measured several years previously.^9^ In order to adjust for missing data, multiple imputation techniques with chained equations were used, assuming missingness to be random, with 100 imputations using all covariates and the CHD mortality outcome variable and time in the study, as previously described.^8^ A sensitivity analysis was conducted to consider whether there was any effect of competing risks due to deaths from causes other than the outcome of interest for each analysis (sudden coronary death and non-sudden coronary death) using the method of Fine and Gray for competing risk regression.^10^

The number of sudden deaths attributable to a high-risk ECG was determined as the number of sudden deaths among men with a high-risk ECG×(adjusted HR−1)/HR.

Finally, exploratory analyses were undertaken to consider whether there were specific ECG abnormalities associated with sudden coronary deaths. The ECG abnormalities considered were T-wave changes (with or without ST depression), Q/QS abnormalities and arrhythmias.

All analyses were conducted using Stata V.14.1 (StataCorp. 2015. Stata Statistical Software: Release 14. College Station, Texas, USA: StataCorp LP).

## Results

Among the 2167 men, there were 262 CHD deaths during a median of 30 years of follow-up, and 719 non-CHD deaths. Of the 262 CHD deaths, 147 were definitely sudden and there were 37 probable sudden deaths. Seventy-eight deaths were definitely non-sudden. Postmortem examinations had been undertaken in 67% of definite sudden, 78% of probable sudden and 23% of non-sudden deaths. Systematic details on interventions during follow-up were not collected, but from the small numbers on whom this information was available, there did not appear to be a difference in coronary artery bypass grafting between those dying from sudden or non-sudden CHD deaths.

Table 2 gives details of the risk factors measured at baseline for each of the four participant groups. There were few differences observed in the risk factors between sudden and non-sudden CHD deaths except for high-risk ECGs. Thus, 34 (18.5%) of the 184 men who suffered sudden deaths had high-risk ECGs at study entry. In contrast, the figures for the 78 non-sudden CHD deaths, 719 non-CHD and 1186 still alive were, respectively, 5 (6.4%), 73 (10.2%) and 89 (7.5%) (p<0.0001 comparing differences across all four groups). As expected, those still alive were noticeably younger at the start of the study than the other three groups.

**Table 2:** Characteristics at baseline, age at end of study and mortality status

		Cause of death*			
	Total	Sudden CHD	Non-sudden CHD	Non-CHD	Alive
All patients	2167	184	78	719	1186
ECG					
Low risk	1966	150 (81.5%)	73 (93.6%)	646 (89.8%)	1097 (92.5%)
High risk	201	34 (18.5%)	5 (6.4%)	73 (10.2%)	89 (7.5%)
Age (years)					
Mean (SD)	46.3	54.7 (6.8)	54.4 (7.8)	53.7 (8.6)	39.9 (11.1)
Age group (years)					
<45	864	19 (10.3%)	8 (10.3%)	100 (13.9%)	737 (62.1%)
45–49	352	21 (11.4%)	11 (14.1%)	110 (15.3%)	210 (17.7%)
50–54	346	48 (26.1%)	16 (20.5%)	147 (20.4%)	135 (11.4%)
55–59	353	52 (28.3%)	22 (28.2%)	198 (27.5%)	81 (6.8%)
≥60	252	44 (23.9%)	21 (26.9%)	164 (22.8%)	23 (1.9%)
Age at event/censoring years					
Mean (SD)	71.9	71.3 (8.7)	72.7 (8.0)	74.1 (10.2)	70.6 (10.6)
Peak flow rate (L/min)					
<430	316	39 (21.2%)	18 (23.1%)	170 (23.6%)	89 (7.5%)
430 – <490	385	42 (22.8%)	20 (25.6%)	163 (22.7%)	160 (13.5%)
490 – <550	584	54 (29.3%)	22 (22.8%)	176 (24.5%)	332 (28.0%)
≥550	878	49 (26.6%)	18 (23.1%)	208 (28.9%)	603 (50.8%)
Missing	4			2	2
Systolic blood pressure (mm Hg)					
Mean (SD)	139	152 (24)	153 (29)	143 (22)	134 (19)
Missing	3				
Body mass index (kg/m^2^)					
Mean (SD)	25.0	26.4 (3.0)	26.2 (2.9)	25.1 (3.0)	24.7 (3.0)
Missing	2	1		1	
Smoking status					
Never	575	24 (13.0%)	14 (17.9%)	106 (14.7%)	431 (36.3%)
Ex-smoker	544	59 (32.1%)	18 (23.1%)	190 (26.4%)	277 (23.4%)
Current smoker	1048	101 (54.9%)	46 (59.0%)	423 (58.8%)	478 (40.3%)
Previous angina					
No	2093	168 (91.3%)	72 (92.3%)	684 (95.1%)	1169 (98.6%)
Yes	74	16 (8.7%)	6 (7.7%)	35 (4.9%)	17 (1.4%)
Cholesterol (mmol/L)					
Mean (SD)	5.91	6.45 (1.29)	6.64 (1.28)	6.04 (1.10)	5.71 (1.13)
Missing	124	10	4	43	67
Fibrinogen (g/L)					
≤3.5	1729	120 (65.2%)	51 (65.4%)	535 (74.4%)	1023 (86.3%)
>3–4	217	36 (19.6%)	16 (20.5%)	91 (12.7%)	74 (6.2%)
>4	126	20 (10.9%)	8 (10.3%)	65 (9.0%)	33 (2.8%)
Missing	95	8	3	28	56
Factor VII (% of standard)					
Mean (SD)	108	118 (30)	115 (30)	111 (27)	104 (26)
Missing	94	7	2	29	56

*Representing 8.5%, 3.6%, 33.2% and 54.7% of the participants, respectively.

CHD, coronary heart disease.

Multivariable associations of risk factors with sudden death are presented in table 3. This confirms the indication in table 2 that the only risk factor for which there is any evidence of a difference between those dying sudden or non-sudden deaths is ECG abnormality, with an adjusted OR of 3.94 (95% CI 1.33 to 11.67) (p=0.006). There was no evidence (see footnote to table 3) that the associations differed according to the interval between the recording of the ECG and death (interaction p value=0.47) although numbers are small. There were similar results in the analysis comparing definite sudden (n=147) with definite non-sudden (n=78) death (ie, omitting probable sudden deaths): adjusted OR 3.95 (95% CI 1.28 to 12.18) (p=0.009).

**Table 3:** Multivariable associations of sudden (n=184) versus non-sudden (n=78) CHD death

	Adjusted* OR	95% CI	p Value
ECG†			
Low risk	1	–	0.006
High risk	3.94	1.33 to 11.67	
Age			
Per 5 year increase	1.00	0.79 to 1.26	0.99
Peak flow rate (L/min)			
<430	0.79	0.33 to 1.92	0.50‡
430 – <490	0.78	0.33 to 1.84	
490 – <550	0.92	0.42 to 2.06	
≥550	1	–	
Systolic blood pressure			
Per 10 mm Hg increase	0.98	0.86 to 1.11	0.73
Body mass index			
Per kg/m^2^ increase	1.02	0.92 to 1.13	0.74
Smoking status			
Non-smoker	1	–	0.90‡
Ex-smoker	2.16	0.85 to 5.49	
Current smoker	1.18	0.47 to 2.93	
Previous angina			
No	1	–	0.78
Yes	0.85	0.28 to 2.57	
Cholesterol			
Per mmol/L increase	0.85	0.66 to 1.09	0.20
Fibrinogen (g/L)			
≤3.5	1	–	0.88‡
>3.5–4	0.82	0.38 to 1.78	
>4	1.10	0.39 to 3.15	
Factor VII (% of standard)			
Per 10 unit increase	1.06	0.95 to 1.19	0.31

*Also adjusted for triglycerides, platelets, white cell count, red blood cell count, haemoglobin, packed cell volume, alcohol use, social class, factor V and factor VIII.

†Unadjusted OR=3.31; age-adjusted OR=3.34. CHD deaths within 10 years (n=61): adjusted OR=2.18 (95% CI 0.38 to 12.62). CHD deaths after 10 years (n=201): adjusted OR=4.95 (95% CI 1.26 to 19.48), interaction p=0.47. Adjusted for age at death: OR=3.97.

‡Trend test.

CHD, coronary heart disease.

Table 4 shows associations separately among all 2167 men prospectively followed for (1) sudden CHD deaths and (2) non-sudden CHD deaths. There was strong evidence for an association of high-risk ECG with sudden death (adjusted HR 1.94 (95% CI 1.31 to 2.89) (p=0.001)), whereas no such association was seen for non-sudden coronary death (adjusted HR 0.55 (95% CI 0.22 to 1.40) (p=0.21)).

**Table 4:** Multivariable association of high-risk ECG with (1) sudden and (2) non-sudden coronary deaths in all 2167 men*

	Sudden death (n=184/2167)			Non-sudden cardiac death (n=78/2167)		
	Adjusted HR	95% CI	p Value	Adjusted HR	95% CI	p Value
ECG						
Low risk	1	–	0.001	1	–	0.21
High risk	1.94	1.31 to 2.88		0.55	0.22 to 1.40	
Peak flow rate (L/min)						
<430	1.40	0.88 to 2.21	0.16†	1.86	0.91 to 3.81	0.045†
430 – <490	1.29	0.83 to 2.00		2.00	1.01 to 3.96	
490 – <550	1.32	0.88 to 1.96		1.58	0.82 to 3.02	
≥550	1	–		1	–	
Systolic blood pressure						
Per 10 mm Hg increase	1.10	1.04 to 1.17	0.002	1.17	1.07 to 1.29	0.001
Body mass index						
Per kg/m^2^ increase	1.09	1.03 to 1.14	0.002	1.07	0.99 to 1.16	0.11
Smoking status						
Non-smoker	1	–	0.037†	1	–	0.22†
Ex-smoker	1.36	0.83 to 2.21		0.69	0.33 to 1.42	
Current smoker	1.65	1.02 to 2.68		1.31	0.67 to 2.55	
Previous angina						
No	1	–	0.034	1	–	0.23
Yes	1.79	1.04 to 3.06		1.74	0.71 to 4.27	
Cholesterol						
Per mmol/L increase	1.15	1.00 to 1.32	0.043	1.32	1.08 to 1.62	0.008
Fibrinogen (g/L)						
≤3.5	1	–	0.007†	1	–	0.025†
>3.5–4	1.47	0.99 to 2.18		1.68	0.92 to 3.06	
>4	1.86	1.09 to 3.15		2.11	0.93 to 4.78	
Factor VII (% of standard)						
Per 10 unit increase	1.05	1.00 to 1.11	0.071	1.01	0.92 to 1.10	0.83

*Also adjusted for current age, triglycerides, platelets, white cell count, red blood cell count, haemoglobin, packed cell volume, alcohol use, social class, factor V and factor VIII.

†Trend test.

From this model, among the 34 men with an ECG abnormality who died suddenly, ∼16 were attributable to the ECG abnormality. Alternatively, of the 184 sudden deaths (ie, sudden and probable sudden), 16 (8.7%) were attributable to high-risk ECGs.

Allowance for missing data gave very similar results and did not change their interpretation. For the competing risk analysis, the association of high-risk ECG with sudden death gave an adjusted HR of 1.88 (95% CI 1.26 to 2.81, p=0.002) with an adjusted HR for the association of high-risk ECG with non-sudden coronary death of 0.49 (95% CI 0.19 to 1.26, p=0.14). The observed HRs of other risk factors previously established as being associated with CHD mortality were broadly similar for sudden and non-sudden CHD, again suggesting that the main distinguishing feature for sudden death is a high-risk ECG.

Considering specific ECG findings, table 5 presents adjusted ORs for T-wave changes: high-risk ECGs other than those showing T-wave changes; and low-risk ECGs (ie, ‘absent’). This suggests that the strongest associations with sudden/non-sudden CHD death are for T-wave changes, although other high-risk ECG abnormalities may contribute (trend test, p=0.004). A second analysis considered T-wave changes, Q/QS abnormalities and arrhythmias jointly in the model. Numbers are again small but the OR of 5.28 for arrhythmias on their own, while not statistically significant, suggests that these may be mainly responsible for any non-T-wave effects.

**Table 5:** Multivariable associations of sudden death versus non-sudden CHD death by T-wave and other ECG changes

	Sudden CHD (n=184)	Non-sudden (n=78)	Adjusted* OR	95% CI	p Value
High-risk ECG abnormality					
No	150	73	1	–	0.004†
Abnormality without T-wave changes	13	3	2.64	0.65 to 10.72	
T-wave changes‡	21	2	5.93	1.22 to 28.81	
T-wave changes					
Absent	163	76	1	–	0.010
Present	21	2	6.09	1.22 to 30.39	
Q/QS pattern					
Absent	180	76	1	–	0.51
Present	4	2	0.50	0.06 to 3.94	
Arrhythmias					
Absent	174	77	1	–	0.075
Present	10	1	5.28	0.61 to 46.00	

*Adjusted for age, peak flow rate, systolic blood pressure, body mass index, smoking status, previous angina, cholesterol, fibrinogen, factor VII, triglycerides, platelets, white cell count, red blood cell count, haemoglobin, packed cell volume, alcohol use, social class, factor V and factor VIII.

†Trend test.

‡Broken down by the presence of ST depression (numbers and adjusted OR (vs no ECG abnormality)):

T-wave alone: sudden deaths=13, non-sudden CHD deaths=1 (adjusted OR 7.26 (95% CI 0.84 to 62.50)).

T-wave and ST depression: sudden deaths=8, non-sudden CHD deaths=1 (adjusted OR 3.50 (95% CI 0.39 to 31.91)).

There were 36 individuals with a T-wave change, Q/QS abnormality and/or an arrhythmia:

23 individuals with a T-wave change (including 3 with a Q/QS abnormality and 1 with an arrhythmia);

3 individuals had a Q/QS abnormality alone; 10 had an arrhythmia alone.

There were three additional individuals with a high-risk ECG abnormality: two with a ventricular condition defect and one with an AV conduction defect.

CHD, coronary heart disease.

Table 6 gives details of the distribution of all abnormalities, singly and combined with each other, among all 2167 men. These support the analyses confined to those experiencing CHD death, showing a trend in the association of ECG abnormalities with sudden death not seen with non-sudden CHD death. Neither Q/QS patterns nor arrhythmias were associated with sudden deaths, though again numbers for these findings were small. The strong associations for T-wave changes are confirmed.

**Table 6:** Multivariable associations of T-wave and other ECG changes with (1) sudden CHD death and (2) non-sudden CHD death*

	Sudden death (n=184)	No sudden death (n=1983)	Adjusted* HR	95% CI	p Value
(1) Sudden death					
High-risk ECG abnormality					
Absent	150	1816	1	–	<0.001†
Without T-wave changes	13	114	1.40	0.78 to 2.52	
T-wave changes‡	21	53	2.60	1.58 to 4.29	
T-wave changes					
Absent	163	1930	1	–	<0.001
Present	21	53	2.52	1.52 to 4.18	
Q/QS pattern					
Absent	180	1959	1	–	0.84
Present	4	24	1.11	0.39 to 3.16	
Arrhythmias					
Absent	174	1903	1	–	0.46
Present	10	80	1.29	0.66 to 2.49	

	Non-sudden CHD death (n=78)	No non-sudden CHD death (n=2089)	Adjusted* HR	95% CI	p Value
(2) Non-sudden CHD death					
High-risk ECG abnormality					
Absent	73	1893	1	–	0.21†
Without T-wave changes	3	124	0.62	0.19 to 2.04	
T-wave changes‡	2	72	0.47	0.11 to 1.99	
T-wave changes					
Absent	76	2017	1	–	0.30
Present	2	72	0.46	0.11 to 1.97	
Q/QS pattern					
Absent	76	2063	1	–	0.28
Present	2	26	2.31	0.51 to 10.53	
Arrhythmias					
Absent	77	2000	1	–	0.17
Present	1	89	0.24	0.03 to 1.81	

*Adjusted for age, peak flow rate, systolic blood pressure, body mass index, smoking status, previous angina, cholesterol, fibrinogen, factor VII, triglycerides, platelets, white cell count, red blood cell count, haemoglobin, packed cell volume, alcohol use, social class, factor V and factor VIII.

†Trend test.

‡Broken down by the presence of ST depression: T-wave alone: sudden deaths=13, non-sudden CHD deaths=1, non-CHD death/alive=40.

T-wave and ST depression: sudden deaths=8, non-sudden CHD deaths=1, non-CHD death/alive=11. For (1) sudden death: adjusted HR=2.31 (95% CI 1.27 to 4.19) (T-wave alone); 3.07 (95% CI 1.39 to 6.78 (T-wave and ST depression). For (2) non-sudden CHD death: adjusted HR=0.33 (95% CI 0.05 to 2.46) (T-wave alone); 0.89 (95% CI 0.11 to 7.07 (T-wave and ST depression).

CHD, coronary heart disease.

Conventional ECG analyses by DAC, based primarily on T-wave changes, significantly predicted the risk of CHD death (as expected) but did not distinguish as clearly as the Minnesota code results between sudden and definite non-sudden death.

## Discussion

Using independent definitions of ECG abnormalities predicting an increased risk of CHD, we found a clear excess of high-risk baseline ECGs in those who later died suddenly compared with those who died non-suddenly from CHD. The main ECG abnormality contributing to the high-risk prediction was T-wave changes. An early report from the Framingham Study was on left ventricular hypertrophy as a risk factor for CHD death, though not necessarily sudden death.^11^ Other abnormalities as risk factors for death, though also not necessarily for sudden death, have included arrhythmias^1^ ^11^ ^12^ such as ventricular premature beats,^1^ ^11^ ^13^ ST-T-wave and/or Q-wave changes,^13–24^ abnormality of ventricular repolarisation and bundle branch block.^25^

There was no evidence that clotting factors can identify those at particular risk of sudden death.

We have defined sudden or probable sudden coronary death as death occurring within 24 hours of the onset of MI, or as death within this time where autopsy findings showed that death was certified as due to CHD, based on the presence of coronary artery thrombosis or severe coronary artery stenosis or occlusion with or without evidence of cardiac muscle infarction. The agonal process of death results in a striking increase in fibrinolytic activity,^26^ which probably accounts for the absence of thrombi in many cases. Our proportion of sudden to non-sudden deaths within the first 24 hours is in accordance with the findings of previous studies.^16^ ^19^ ^27–29^

A strength of our study is the prior availability from another study of detailed ECG findings associated with CHD, thus providing independent definitions of abnormalities in relation to later death from CHD (though not necessarily sudden death). Evaluations of end point events and decisions on intervals between onset of symptoms and death were also made independently.

Our results suggesting T waves as a risk factor for sudden death have been clear, significant and consistent in different analyses. However, there are several questions to be answered before our results could be considered for application in practice. First, the data were collected >30 years ago, since when there have been major changes in preventive and clinical cardiology. Could these changes have altered or abolished the associations we found? Second, the data come from a single study in which the numbers of those with differing ECG findings and also of those who died suddenly or non-suddenly are obviously not large enough to suggest applying our results in practice, though they have been sufficient in our study at least to raise the question. Third, several of the original participants in NPHS-I were elderly when they died, so that multiple pathologies have to be considered in assigning the causes of death. We believe we have minimised inaccuracies by collecting information from all available sources, including pathological details from the many autopsies carried out (see below). Fourth, how accurate were classifications into the sudden, probable and non-sudden groups? We had stringent requirements for classifying deaths as sudden or non-sudden. The high proportion of autopsies in the probable group (78% compared with 23% in the non-sudden group) suggests that the majority of these deaths were unexpected and indeed sudden, so that combining them with sudden deaths was justified, and that there will have been few misclassifications of the intervals to death. The involvement of coroners' officers was particularly helpful in recording information on timings. In fact, any mistakes over causes of death or intervals will have reduced rather than have exaggerated the strength of the associations we have described. Finally, the concomitant contribution of hypertension or left ventricular hypertrophy could not be assessed with any certainty because of the omission of men with more serious hypertension at recruitment, though an advantage is that we have assessed the effects of ECGs largely independent of any associations with blood pressure.

In conclusion, among a group of men who had not previously experienced major episodes of CHD but who subsequently died from it, there was strong evidence that high-risk ECG changes, mainly T-wave abnormalities, differentiated between those who later died sudden deaths and those who survived for >24 hours. Further research on this potentially important topic should be a high priority. As a start, case–control studies would avoid the drawbacks of our prospective study and could produce early results indicating whether or not it would be worth carrying out more elaborate and long-term studies. Research should also assess whether reading ECGs in day-to-day clinical practice also indicates T-wave changes as indicators of an increased risk of sudden death. If so, reliance on the Minnesota code could be avoided.
